# Stealthy and hyperuniform isotropic photonic band gap structure in 3D

**DOI:** 10.1093/pnasnexus/pgae383

**Published:** 2024-09-06

**Authors:** Lukas Siedentop, Gianluc Lui, Georg Maret, Paul M Chaikin, Paul J Steinhardt, Salvatore Torquato, Peter Keim, Marian Florescu

**Affiliations:** Department of Physics, University of Konstanz, Konstanz 78457, Germany; Advanced Technology Institute and School of Mathematics and Physics, University of Surrey, Guildford, Surrey GU2 7XH, United Kingdom; Department of Physics, University of Konstanz, Konstanz 78457, Germany; Department of Physics, New York University, New York, NY 10003, USA; Department of Physics, Princeton University, Princeton, NJ 08544, USA; Department of Physics, Princeton University, Princeton, NJ 08544, USA; Department of Chemistry, Princeton University, Princeton, NJ 08544, USA; Princeton Materials Institute, Princeton University, Princeton, NJ 08544, USA; Program in Applied and Computational Mathematics, Princeton University, Princeton, NJ 08544, USA; Max-Planck-Institute for Dynamics and Self-Organization, Göttingen 37077, Germany; Institute for the Dynamics of Complex Systems, University of Göttingen, Göttingen 37077, Germany; Institute for Experimental Physics of Condensed Matter, Heinrich-Heine-Universität Düsseldorf, Düsseldorf 40225, Germany; Advanced Technology Institute and School of Mathematics and Physics, University of Surrey, Guildford, Surrey GU2 7XH, United Kingdom

**Keywords:** stealthy, hyperuniform, isotropic photonic band gap

## Abstract

In photonic crystals, the propagation of light is governed by their photonic band structure, an ensemble of propagating states grouped into bands, separated by photonic band gaps. Due to discrete symmetries in spatially strictly periodic dielectric structures their photonic band structure is intrinsically anisotropic. However, for many applications, such as manufacturing artificial structural color materials or developing photonic computing devices, but also for the fundamental understanding of light-matter interactions, it is of major interest to seek materials with long range nonperiodic dielectric structures which allow the formation of *isotropic* photonic band gaps. Here, we report the first ever 3D isotropic photonic band gap for an optimized disordered stealthy hyperuniform structure for microwaves. The transmission spectra are directly compared to a diamond pattern and an amorphous structure with similar node density. The band structure is measured experimentally for all three microwave structures, manufactured by 3D laser printing for metamaterials with refractive index up to n=2.1. Results agree well with finite-difference-time-domain numerical investigations and a priori calculations of the band gap for the hyperuniform structure: the diamond structure shows gaps but being anisotropic as expected, the stealthy hyperuniform pattern shows an isotropic gap of very similar magnitude, while the amorphous structure does not show a gap at all. Since they are more easily manufactured, prototyping centimeter scaled microwave structures may help optimizing structures in the technologically very interesting region of infrared.

**Significance Statement**

Photonic integrated circuitry promises faster and less dissipative processing of information and is steadily replacing its electronic analog. The preponderance of previous work has focused on the use of photonic crystals that prohibit the propagation of electromagnetic waves within a band gap, thereby allowing for the control of wave transport. Here, we demonstrate, for the first time, that 3D disordered “stealthy hyperuniform” materials, a special class of correlated amorphous systems, allow for the formation of sizeable isotropic photonic band gaps. Such materials with novel optical properties enable the manipulation of light with exquisite control. They also are expected to have applications in the design of structural colors and the control of the propagation of sound waves.

## Introduction

The manipulation of light propagation by employing periodic dielectric structures is widely used in technology, e.g. in dielectric mirrors and antireflection coatings. For this purpose, 1D periodic structures reflect or transmit only a narrow part of the electromagnetic spectrum due to Bragg scattering. Bragg scattering strongly depends on the orientation of the structure with respect to the incident wave and is thus intrinsically anisotropic. The generalization to 3D structures leads to so called photonic crystals with stop bands for the propagation of light in various directions of the given Brillouin zone in close analogy to electronic band gap formation (1–4). Here, the typical length scale is given by the Bragg condition, thus the dielectric metamaterial is structured on a scale comparable to the electromagnetic wavelength: centimeter range for microwaves and sub-micron range for visible light.

2D photonic structures exhibiting a complete photonic band gap, for both TE and TM polarizations, are rather challenging to realize. This is because, even for periodic structures, the architectures needed are rather different, TM-polarization photonic band gap opening very easily in isolated scatterer architectures, whereas the optimal favored architecture for opening of TE-polarization band gaps consists of connected dielectric network structures. Complete bandgaps in 2D can be opened in structures which reach a compromise between the two architectures and consist of dielectric scatterers connected by narrow dielectric veins (5–7) but the largest complete gaps reach a rather modest size of just 15% of the midgap frequency for silicon-air index of refraction contrast (6). In contrast, 3D photonic structures based on a diamond-network architecture are naturally adapted for the opening of complete photonic band gaps and can reach band gaps of about 30% of the midgap frequency for the same index of refraction contrast (8). However, as is the case with all periodic structures, the high-symmetry directions in the underlying FCC lattice, induces strongly anisotropic photonic band gaps.

For technological applications but also from a fundamental point of view it is of enormous interest to find materials with isotropic photonic band gaps where the photonic density of states disappears in all directions. It has been long argued that isotropic band gaps will form in dielectric metamaterials whose structure is itself isotropic (9). So called hyperuniform structures (7), where the structure factor vanishes in the long-wavelength limit were tested to have a band gap in 2D for microwaves (10–12). For a *D*-dimensional point pattern in a *D*-dimensional spherical sampling window with radius *R*, hyperuniformity is defined as the number variance of points contained within the spherical window, σ(R) when averaged over all possible positions of the window within the domain being considered. The point pattern is hyperuniform if σ(R) grows as $R^{D-1}$; that is the number variance is proportional to the surface area of the sampling window rather than its volume as is the case of e.g. Poisson point patterns (13, 14). Hyperuniform point patterns include all photonic crystals, quasicrystals and a subset of disordered structures. They possess zero density fluctuations on infinite length scales within the structure so their structure factor S(k) vanishes for k→0.

For relatively large refractive indices, Muller et al. and Aeby et al. have demonstrated 3D emergent isotropic band gaps in the near infrared (15–17). Their structures are based on disordered jammed packings that are hyperuniform and nearly stealthy. Besides hyperuniformity, the optimization of short-range order to tailor Bragg scattering at the Brillouin zone is of key importance, too (18, 19). Furthermore, internal Mie-resonances within the high-index material (spheres or cylinders) affect the photonic density of states (20–22). To characterize the short-range order, Sellers et al. (23) introduced the concept of local self-uniformity in cylinder based structures, where Mie-resonances within the cylinders have to interfere constructively with the structural arrangement of the cylinders. 3D Amorphous structures with diamond-like local tetrahedral order were investigated numerically (24) and experimentally in the microwave regime (25) showing photonic band gaps which were compared to diamond structures. It is important to stress the differences in the size of the PBG when comparing disordered nonstealthy hyperuniform structures to their stealthy hyperuniform counterparts as the system size increases. A comprehensive 2D study of disordered structures that range from nonhyperuniform to standard hyperuniform and stealthy hyperuniform ones revealed that the apparent PBGs rapidly close as the system size increases. This is for all disordered networks under consideration, except for the stealthy hyperuniform structures where the PBG persists (26). For the same reasons, we expect that 3D stealthy hyperuniform dielectric networks have such PBG superiority.

**Fig. 1.**
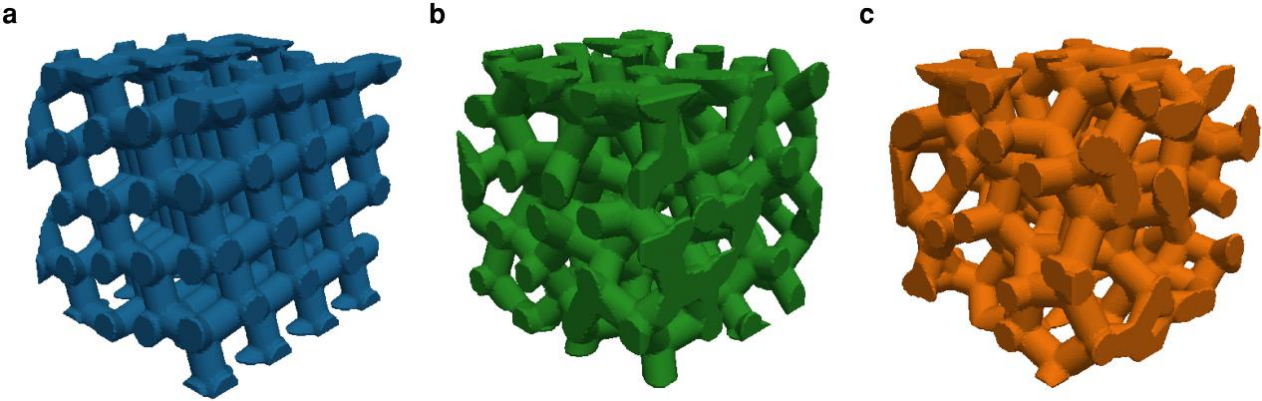
Excerpts of the three cylinder model structures under investigation. a) A diamond pattern, possessing a well-known but anisotropic band structure, b) the optimized stealthy hyperuniform pattern with isotropic band structure, c) an isotropic pattern generated from an amorphous glassy seed pattern from Monte Carlo simulations for comparison. The thickness of the rods is 0.3a.

In the present work, we investigate three photonic structures composed of an interconnected tetravalent network of cylinders (i) of an anisotropic diamond lattice, (ii) an optimized isotropic and stealthy hyperuniform structure, and (iii) an isotropic network structure constructed from a glassy, random hard sphere packing seed pattern obtained from computer simulations. Figure 1 visualizes the three structures with a) the diamond structure in blue, b) the stealthy hyperuniform pattern in green and c) the disordered pattern in red. For the diamond structure (blue), we expect a band structure but being strongly directional. The isotropic glass structure (red) is constructed for comparison and as reference for the optimized hyperuniform structure (green). In analogy with periodic structures, we define a length scale $a=L/\sqrt[{3}]{N}$ such that an N-point pattern in a cubic box of side length *L* has a scatterer density of $1/a^{3}$. Samples are shown in a cube of L=4×a side length, while the investigated structures in the spectrometer have a side length of L=10×a.

The optimized hyperuniform structure was constructed by combining continuous random networks (CRN) inspired by models of amorphous silicon (24, 27, 28) and hyperuniformity concepts (7, 13, 14, 23). The CRN structures are generated by annealing a completely random four-fold coordinated network using the Wooten–Winer–Weaire (WWW) algorithm (29, 30). The algorithm proceeds by introducing coordination-preserving Stone–Wales defects at random positions in the structure followed by subsequent relaxation of the structure with a Keating potential to minimize the spread in the distributions of the next-neighbor particle distance and the angles made by the tetrahedral bonds in the network (typical values for standard deviations of the distributions for optimized CRN are around $\sigma_{d}\approx 5\%$ and $\sigma_{\theta }\approx 9\%$) (23, 31). While the continuous random network structures obtained through the WWW algorithm present well-defined short-range order, they are not yet hyperuniform. For photonic applications (7), we are interested in an optimized subcategory of hyperuniform structures, namely “stealthy” hyperuniform structures (13, 14).

**Fig. 2.**
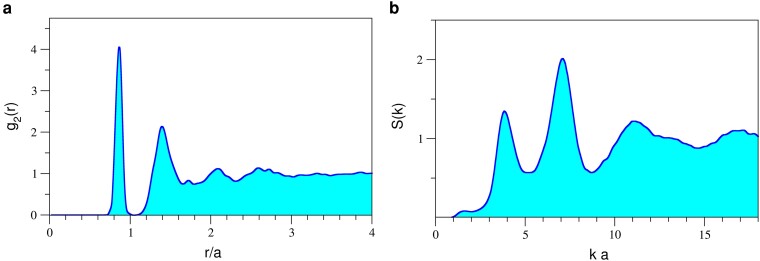
Two-point correlation function a) and structure factor b) for the optimized stealthy hyperuniform point pattern constructed from an N=1,000 CRN model, with χ=0.03. For stealthyness, the structure factor is forced to zero for ka<1.

**Fig. 3.**
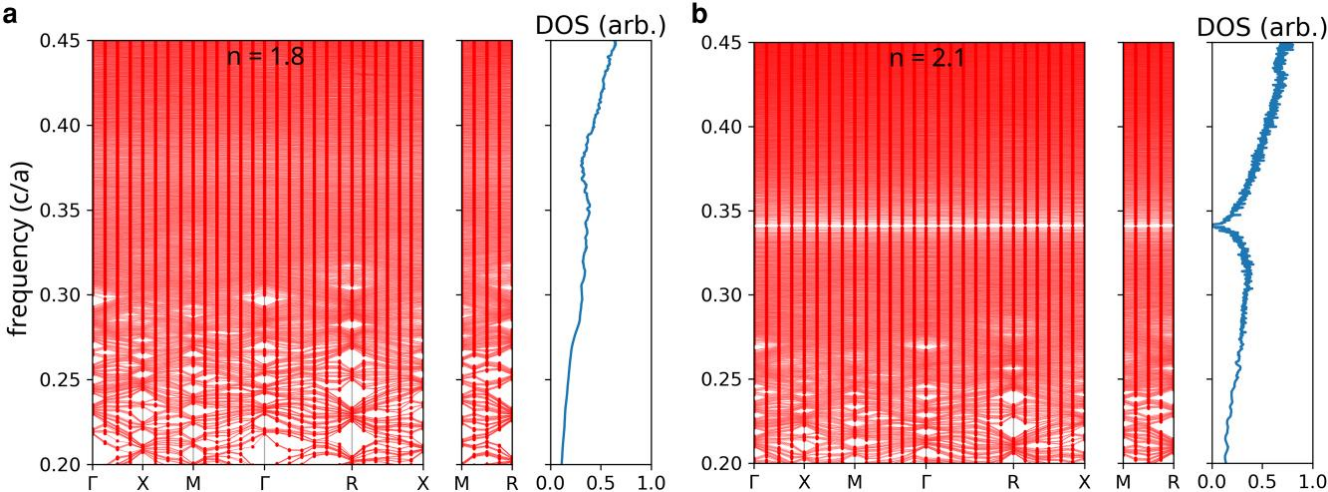
Band structure for dielectric-rod decorated optimized hyperuniform structures for contrast of the indices of refraction corresponding to a) composite of alumina n=1.8 and b) composite of titania n=2.1. While for n=1.8 only a dip in the DOS is visible, a small but complete and isotropic band gap opens up for n=2.1. The band structure and density of states are calculated for a sample hyperuniform network of 1,000 vertices decorated with dielectric rods of radius r/a=0.3 contained in a $[10\times a{]}^{3}$-supercell.

For stealthy hyperuniform point patterns, the structure factor S(k) is statistically equal to zero for a finite range of wave numbers smaller than a certain critical wave vector $k_{C}$, i.e. $S(k<k_{C})=0$. The stealthiness parameter χ=M/3N is defined as the ratio between the number of *k* vectors for which the structure factor is constrained to vanish, *M*, and the total number of *k* vectors associated with the pattern, 3N (with *N* the number of points in the pattern). Here, we employ continuous random networks generated by using the WWW algorithm; the structures are subsequently made hyperuniform by forcing the structure factor at a fixed number of *k* values to vanish. To maintain the well-defined short-range order associated with the CRN, we employ only a small number of wavenumbers. In Fig. 2, we present the two-point correlation function, $g_{2}(r)$ and the structure factor S(k), for an N=1,000 optimized stealthy hyperuniform pattern. Here, we enforce the structure factor to vanish for the smallest 100 *k* values around the origin (i.e. χ=100/3,000≈0.03). In Fig. 3, we present the corresponding band structure and density of states calculations for a structure built by decorating the hyperuniform point pattern with dielectric rods of various indices of refraction. The original stealthy hyperuniform point pattern is generated under periodic boundary conditions, and the band structure was calculated using a supercell approximation. The refractive indices were chosen to map the experimental accessible ones. While for n=1.8, only a dip in the density of states around f=0.375 (in units of c/a) is visible, for n=2.1 a complete and isotropic gap opens up around f=0.34. The width of the gap is 0.6%. We note that for higher refractive indices, e.g. n=3.4 the gap width increases up to 14% and is centered at f=0.24c/a (not shown here). As shown in Fig. 3b, the band gap is independent of the orientation of the wave vectors and it is isotropic as intended.

## Experimental and numeric results

Macroscopic structures given by cubes with 100 mm side-length and operational wavelengths of about 30 mm where manufactured, corresponding to frequencies in the 10 GHz range. This is inspired by the seminal work of Yablonovitch (8) and previous applications to photonic amorphous diamonds (24, 25). All three structures where realized with a 3D printer using selective laser sintering (SLS) of a compound material. This compound material consists of a polymer (Nylon) with additives of high refractive index materials as ${\text{Al}}_{2}{\text{O}}_{3}$ and ${\text{TiO}}_{2}$. The mass ratio of the oxides in Nylon were increased up to a value until SLS printing failed to produce a mechanical stable object. For ${\text{Al}}_{2}{\text{O}}_{3}$, a refractive index of about n=1.8 was accessible, while for ${\text{TiO}}_{2}$ we manufactured samples with n=2.1 in the given frequency range of the microwaves. Details on the printing procedure are given in.

**Fig. 4.**
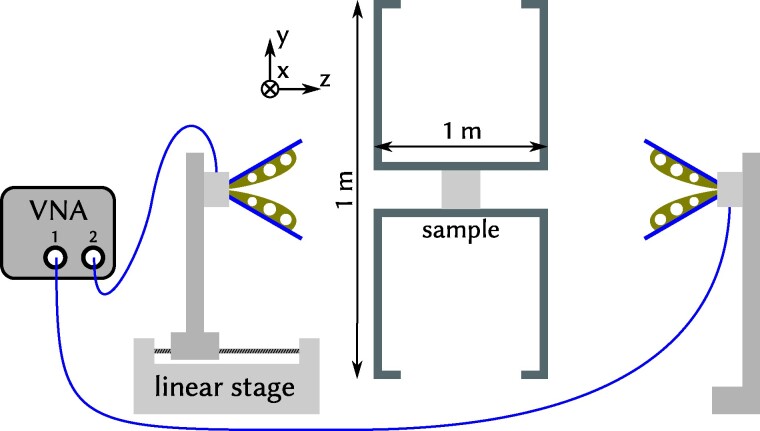
Sketch of the setup to measure the transmittance through macroscopic photonic structures. It consists of a vector network analyzer (VNA), two horn antennas, and a 1m wave-guide with a 10cm quadratic aperture in a $1\;m^{2}$ beam block. The samples are put in the middle of the waveguide. The position of the left antenna relative to the waveguide can be adjusted with a linear stage in the range of 0.1 to 0.38 m, the distance of the right antenna to the waveguide is 0.4 m. One hundred measurements are averaged at different positions of the left antenna.

**Fig. 5.**
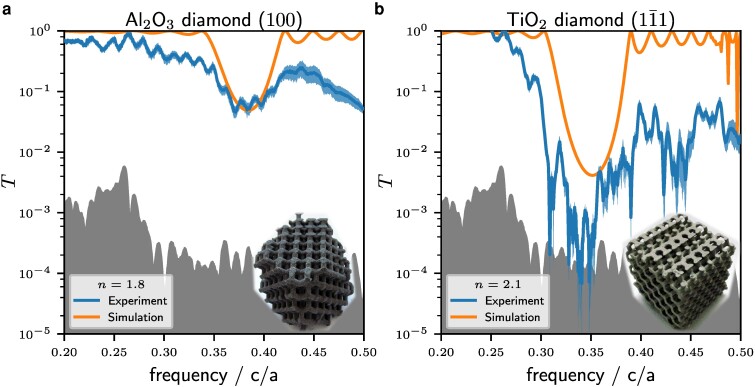
Results of transmission measurements for two macroscopic realizations of the metamaterial of diamond. Spectra are taken in two different directions of the lattice and for two different refractive indices n=1.8 and n=2.1. The cubic samples have a side-length of 10 cm. The ${\text{Al}}_{2}{\text{O}}_{3}$ samples suffer from mechanical damage due to their brittleness, as exemplary shown in the inset image of the diamond in a). The peak position as well as the depth of the peak coincide well for a) Al_2_O_3_ in (100) and b) TiO_2_ in (1$\bar{1}$1) direction.

The transmittance of the samples is measured by placing the sample in a wave guide that just fits the sample, as sketched in Fig. 4. Two antennas emit respectively receive linear polarized electromagnetic radiation from at least 0.05 to 13.5GHz, as controlled by a vector network analyzer. More details about the setup are given in Measurement of transmission spectra section. First of all, the dark spectrum of the setup is shown as the shaded gray area at the bottom of each spectrum. It is the experimentally minimal detectable signal of the given device. It reflects the dynamic range of the vector network analyzer, the efficiency of the antenna and the mode-structure of the wave-guide. As function of frequency, the dynamic range of the setup is determined to be 30–40 dB. Images of the laser-sintered diamond structures are shown in the insets of Fig. 5 along with the measured transmittance curves (blue) and the numerically calculated ones (orange) using the software package meep, as described in Simulations of transmission spectra section. We present data from the diamond structure made of ${\text{Al}}_{2}{\text{O}}_{3}$ in (100) direction while the ${\text{TiO}}_{2}$ is presented in ($1\bar{1}1$) direction. Both structures acts as proof of principle for the experimental setup. The positions and depth of the gaps coincide very well in both directions for the given refractive indices. Note, that for the ($1\bar{1}1$) direction with n=2.1, the transmission is reduced by almost three orders of magnitude at a frequency of f=0.34c/a. However, beside the gap in transmission, the experimental data (blue/dark in b/w) hardly recover the maximal transmission on the high-frequency side, while the simulations (orange/bright in b/w) do. This might be attributed to three distinct effects: (i) The experiment contains some absorption which is absent in the simulation. However, the refractive index of Nylon, ${\text{Al}}_{2}{\text{O}}_{3}$, and ${\text{TiO}}_{2}$ was measured to be frequency independent in the region of interest (32). Since absorption takes place at electronic resonances where ∂n/∂f≠0 and since the long-wavelength limit transmission almost recovers T=1, absorption is neglected in the following, in line with (25). (ii) The horn-antennas transmit (or receive) signals only within a finite angle. While the microwave will be reflected within the wave-guide on the metallic surface, losses are expected via radiation beside the optical axis in the region between the wave-guide and the antenna. This is different in the simulation, where the flux planes detects radiation in and from all directions and no loss can appear due to metallic boundary conditions up to the position of the detection area. In short, the FDTD simulations effectively measures the hemispherical reflection/transmission, directly next to the sample, while the experiment is sensitive to directional reflection/transmission at a finite distance, see (Simulations of transmission spectra section). (iii) The antennas in experiment are linear polarized and do not detect electromagnetic waves which are depolarized due to scattering, while the detection in the FDTD simulation is insensitive to depolarization. Scattering is stronger for higher frequencies thus (ii) and (iii) cause the transmission better to recover T=1 below the gap than above the gap for both, the (100) and ($1\bar{1}1$) direction of the diamond.

To gain insight about the sensibility of the device with respect to the local geometry and resonances within the wave-guide, the left antenna was mounted on a linear stage (along the optical axes), and data were taken at about 100 different positions of the antenna. The shaded blue region of the experimental data denotes the standard deviation from averaging about this 100 positions. As can be seen in Fig. 5, the measurement is rather stable with regards to linear translation of the antenna.

**Fig. 6.**
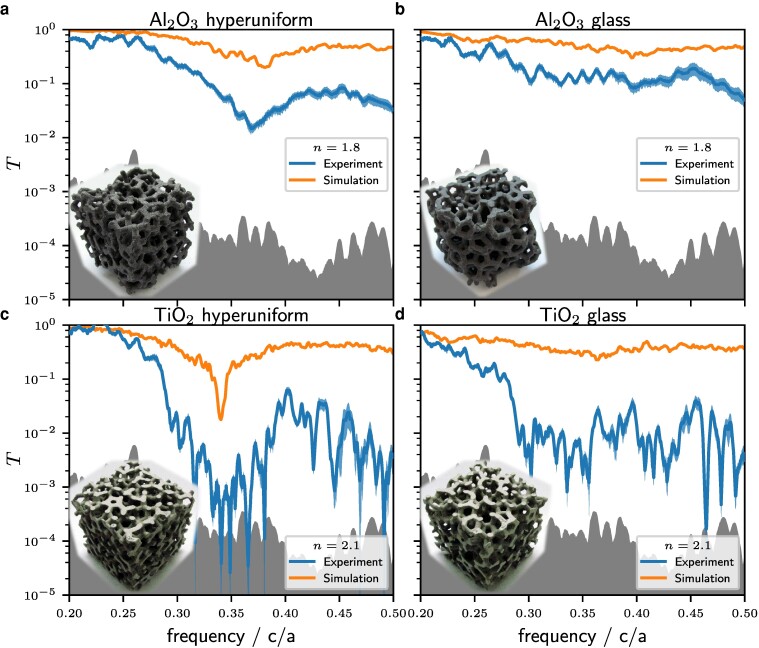
Results of the macroscopic realizations for two different refractive indices n=1.8 (upper row, a) & b)) and n=2.1 (lower row, c) & d)) for the stealthy hyperuniform structure (left column, a) & c)) and the amorphous sample constructed from structural glass data (right column, b) & d)). The cubic samples in experiment have a side length of 10cm=10a.

The structure of interest is the stealthy hyperuniform one, shown in the left column of Fig. 6. For n=1.8 (upper row, a) & b)) a dip at f=0.37 shows up while at n=2.1 (lower row, c) & d)) the gap is positioned at f=0.34 and is 20dB for the FDTD simulations and even 30dB for the experimental data. The shaded blue region, indicating the averaging about 100 different positions, is of similar size compared to the diamond structure. The local “spikes” visible in the experimental transmission curves of Figs. 5 and 6 are not averaged by translation, thus we attribute them to resonances within the sample (e.g. at imperfections and surface roughness) at the given finite size of 10 nodes in linear dimensions. Note, that spikes appear enhanced on a log-scale in the minima of transmission. The right column shows an isotropic glass as reference structure which lacks a photonic band gap. Again, the reduced transmission on the high-frequency side is more pronounced in the experiment with directional sensitive detection compared to the FDTD simulations with hemispherical sensitive detection. While isotropic per definition, spikes in transmission are visible, again attributed to local resonances within the finite sized sample. While both structures are tetravalent and look similar by eye, the standard deviation of the node distance varies significantly: it is 0.035a for the hyperuniform pattern and 0.09a for the glass pattern. The same holds for the standard deviation of angle distribution of the network which is $9^{\circ }$ of the stealthy hyperuniform structure and $14^{\circ }$ for the glass pattern. The optimized stealthy and hyperuniform pattern allows for destructive interference but—unlike in periodic structures—in all spatial directions.

**Fig. 7.**
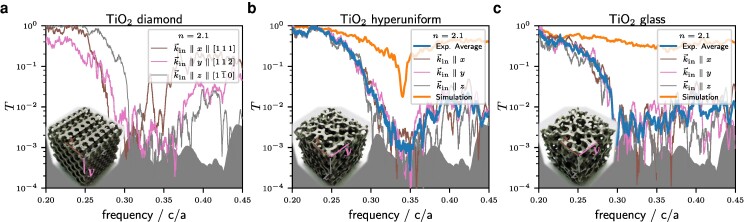
Measurements of three different orientations of a) the diamond, b) stealthy hyperuniform structure, and c) the isotropic glassy structure relative to the incoming wave-vector ${\vec{k}}_{in}$. The spikes show an individual structure (enhanced by the log-scale in the minima) and are of comparable size for each structure and orientation. While the spectra of the diamond varies significantly with orientation as expected, the gap position and depth is the same for the stealthy hyperuniform structure, indicating isotropy. The glass structure is isotropic, too, but only shows an edge.

To check for isotropy, all structures were additionally measured in three different orientations of the wave vector with respect to the cubes. Figure 7 shows the transmission for the different orientations relative to the incoming wave vector. The diamond a) is taken as reference showing significant anisotropy. Different orientations show individual spike-patterns for all three structures. However, in contrast to the diamond, the position and depth of the minima are similar for the stealthy hyperuniform pattern b). Beside the spike pattern, this indicates isotropy. The black curve is the average of orientations showing less of the spike structure but a well-defined minimum: the decrease in transmission is 30dB recovering more than 10dB above the gap. The glass c) is isotropic but shows a edge instead of a gap. The orientation-average indicates a decrease in transmission for higher frequencies due to scattering.

## Conclusion

Three different photonic structures in the microwave range were constructed by 3D laser printing of a compound material. The structures of interest is a stealthy hyperuniform one which was optimized to show an isotropic photonic band gap for sufficiently large refractive index. A diamond structure with well-known but anisotropic band structure and a amorphous structure constructed from a 3D glass former were investigated as reference. For all three structures, the transmission spectra were measured in a wave-guide, using horn-antennas and a vector network analyzer. The spectra were compared to finite difference time domain calculations (FDTD), where Maxwell’s equations are solved numerically on a grid and to a priory band structure calculations.

The diamond structure shows a clear gap in the (100) and ($1\bar{1}1$) direction, depending on orientation and increasing with refractive index, as expected. The stealthy hyperuniform structure shows a dip in transmission for $n_{\exp}=1.8\pm 0.35$ and an isotropic band gap for $n_{\exp}=2.1\pm 0.38$: the transmittance is reduced by three orders of magnitude and recovers more than one order of magnitude back above the gap in the high-frequency range. Thus it performs almost as well as the diamond along a symmetry axis but being isotropic. Furthermore the gap opens at a moderate refractive index—stealthy hyperuniformity is key to produce a gap for parameters more easily found in materials in the microwave or optical range.

The reduction in transmission of up to three orders of magnitude in the gap is true for rather small samples with ten nodes in linear dimensions. It can be significantly enhanced since doubling the structure in length will square the result. The small size on the other hand causes an individual spike structure in the spectra even for the amorphous sample which averages for various orientations of the cubes but not translations of the antenna. For both nonperiodic structures, the hyperuniform and the glassy one, the transmittance does not recover $10^{0}$ in the high frequency range, neither in the experimental data, nor in the simulations. For the periodic structure, only the simulation recover full transmission above the gap. The reduced transmission on the high-frequency side is attributed to the residual disorder of the structures which scatter intensity into the 4π space, not captured by the detector. An analysis of scattering by defects in the small wavelength spectrum is beyond the scope of this article. For woodpile structures, this is given in (33). While looking very similar by eye, the completely disordered amorphous structure does not show a gap at all but an edge, as expected.

## Methods

### Sample production with 3D printing

Selective laser sintering (SLS) offers the possibility to construct samples from in principle any powder (partially) composed of meltable grains. The only restriction is the interconnected morphology of the structure, providing the chance to produce photonic structures of any kind. The refractive index of the structure is tunable by carefully choosing the powder constituents. The 3D printer used in this work is a Sintratec Kit (34). The meltable grains are made of Nylon (PA12). They are around 50μm in size and the melting point of Nylon is approximately 185^∘^C (34). Those grains were mixed with grains of (${\text{Al}}_{2}{\text{O}}_{3}$) or (${\text{TiO}}_{2}$) to increase the refractive index of the compound material above that one of PA12. Samples with a maximum side length of 100 mm can be realized with this specific machine.

Printing protocols of powders with the two different additives aluminum oxide (${\text{Al}}_{2}{\text{O}}_{3}$) and titanium dioxide (${\text{TiO}}_{2}$) are developed similar to the procedure described in references (25, 35). The aim is to print structures of a wide range of refractive indices. The base material Nylon (PA12), (${\text{C}}_{12}{\text{H}}_{2}3\text{NO}{)}_{n}$) has a refractive index $n_{\mathrm{PA}12}\approx 1.5$ in the frequency regime of interest 0.5 to 18 GHz. Undyed PA12 (AdSint PA12 L nat, Advanc3D Materials GmbH) with about 40μm grain size darkened with a small amount of carbon black to aid laser absorption is used for ${\text{Al}}_{2}{\text{O}}_{3}$ powder mixtures and gray material from Sintratec with about 50μm grain size is used for the ${\text{TiO}}_{2}$ mixtures. All materials have a small loss tangent (25) in the GHz-regime thus absorption is neglected in the accompanying numeric investigations. ${\text{Al}}_{2}{\text{O}}_{3}$ has $n_{{\mathrm{Al}}_{2}O_{3}}\approx 3$ (36) and material with a grain size of 50μm is used (product number 007–0160, Final Advanced Materials). ${\text{TiO}}_{2}$ has an refractive index of $n_{{\mathrm{TiO}}_{2}}\approx 10$ (37, 38) in the bulk. It is found that the commonly available nanosized ${\text{TiO}}_{2}$ of particle sizes around 500 nm tends to form agglomerates that are difficult to compound with the PA12. It is also found that it changes the pouring behavior of the powder such that dense and mechanically stable final materials are not readily achievable. Kronos 3,025 (rutile, KRONOS Worldwide, Inc.) is found to consist of a broad range in size-distribution of the grains. The nanoparticles are washed out via sedimentation (1 kg Kronos 3,025 stirred in 5 l water, 5 min sedimentation time, 5 repetitions). The remaining particles are dried and sieved. Grains in the size range 20μm<d<180μm were selected for the compound material. For the final compounds, mass ratios of 4 : 1 $m_{{\mathrm{Al}}_{2}O_{3}}$ : $m_{\mathrm{PA}12}$ and 14.7 : 1 $m_{{\mathrm{TiO}}_{2}}$ : $m_{\mathrm{PA}12}$ where used, as well as a negligible amount of carbon black to increase the absorption of the laser for melting (≤1 g per 1,000 g compound).

For microwaves with wavelength in the cm range, the refractive index of the metamaterial is the average of the refractive indices $n_{i}$ of the components *i* weighted by their volume filling fractions $\phi_{i}$ and reads $n_{\mathrm{eff}}=\sum_{i}\phi_{i}n_{i}$. However, since the compound material has inclusion of air, this does not give a reliable effective refractive index, and we measure it instead with a time of flight based measurement: the time difference Δt between two microwave signals reflected at the front and at the back of the structure of length *L* relates to the (average) refractive index $n_{\exp}$ of the structure via $n_{\exp}=c\Delta t/2L$. Δt is obtained by measuring the peak distance of the Fourier-transformed ${\text{S}}_{11}$-signal of the vector network analyzer.

The thickness of the rods is 0.3a, optimized by parameter sweep in FDTD simulations for the spectra of the stealthy hyperuniform structure for the given refractive index. Accordingly, the volume fractions are 33, 32, and 31% for the diamond, the stealthy hyperuniform, and the glass structure.

### Measurement of transmission spectra

In order to measure the transmittance through a photonic structure, a two port network is set up with a vector network analyzer. The spectra in Figs. 5 and 6 were measured with a vector network analyzer (VNA) ZNB40 from Rohde & Schwarz, frequency range 0.05 to 40GHz, while the spectra in Fig. 7 were measured with HP-8719D 0.05 to 13.5GHz. Two identical antennas (Aaronia PowerLOG 70,180, 0.7 to 18GHz) emit (and receive) the signal and the sample is placed in between both antennas in the wave-guide. The wave guide of width 10a and a length of 1m is used to reduce scattering of the electromagnetic wave in all directions beside the optical axis. It includes metallic shield acting as aperture of 1m×1m at both sides of the wave-guide as sketched in (Fig. 4), to avoid a short-cut of the electromagnetic wave bypassing the sample. The antennas emit electromagnetic waves of linear polarization which defines the *y*-axis and the wave-guide is oriented along the *z*-axis. To calculate the transmittance, the transmission coefficients with a sample in the guide is measured and squared to get the intensity. This intensity is normalized by the corresponding value of the spectrum without sample in the guide, to eliminate the specific features of the antenna and the wave-guide. To further average interference effects within the wave guide, a linear stage is used that positions one antenna within a 250mm range. The total transmittance is taken as the average measurements of transmission $S_{12}=S_{21}\;:\;=t$ for various different positions of the emitting antenna. The spectra presented in Figs. 5 and 6 are averaged about a hundred different positions. Based on the finite size of the antenna and using the wave-guide with shield, only a small fraction of the whole half space is detected. Thus, *R* measures the *directional* reflection and *T* measures the *directional* transmission.

### Refractive index

By applying the Fourier transform to a reflection *S*-parameter ($s_{11}$ or $s_{22}$) as a function of frequency one can study its behavior in the time domain. The maximum frequency of the VNA dictates the time resolution and the frequency resolution dictates the maximum time that can be studied due to the inverse nature of the Fourier transform. In doing so, the impedances of the system can be evaluated. Any peak in the time domain indicates an impedance step like in time of flight measurements. Since the geometry of the setup and the sample is well known, the spectrally averaged refractive index $n_{\mathrm{eff}}$ via $n_{\mathrm{eff}}=\frac{ct}{2L}$ can be extracted, where *L* is given by the linear size of the sample and the corresponding peaks in the time domain are the discontinuities in impedance at the front and back side of the sample. The factor of 2 arises since the signal runs back and forth to the VNA. Together with the volume fraction, $n_{\mathrm{eff}}$ gives the high refractive index with the uncertainty $\delta n=n(\frac{\delta t}{t}+\frac{\delta L}{L})$. This method provides a constant value for *n* and dispersion is not accounted for. The refractive index of the compound material of PA12 with (${\text{Al}}_{2}{\text{O}}_{3}$) was determined to be n=1.83±0.35 and that of PA12 with (${\text{TiO}}_{2}$) to 2.15±0.38. Since the uncertainty is large, the spectra are compared with numeric results of transmission spectra of the diamond lattice, where the refractive index was varied in steps of Δn=0.1 (Simulations of transmission spectra section). Best agreement was found for $n_{Al_{2}O_{3}}=1.8$ and $n_{TiO_{2}}=2.1$.

### Simulations of transmission spectra

Finite difference time domain (FDTD) simulations with the ab initio implementation of the software package MIT electromagnetic equation propagation (meep) (39) are performed in order to simulate the transmittance through a well-defined structure. The structure is represented as a spatially varying refractive index $n(\vec{r})=\sqrt{\varepsilon (\vec{r})}$ relative to the refractive index of the vacuum n=1. Dimensionless length units denoted by 1 a are chosen. The structure of interest is sized to a cube with 10 a side length as in experimental investigations. A 2 a cladding of air is added in *z*-direction at both sides in which the source plane and flux plane lies which detects the passing intensity. Another 2 a cladding of absorbing material is added in *z*-direction at both sides. This ensures that radiation is not scattered back onto the sample. Metallic boundary conditions are chosen, resembling the experimental setup. The software then divides the geometry into a grid whereon the electromagnetic field is calculated for each timestep based on the previous fields strengths.

The source is set to emit a plane wave with a Gaussian distribution in frequency. The reflection and transmission can be extracted as function of frequency by Fourier transforming the signal at the respective flux plane. The frequency is given in units of speed of light per length and denoted as c/a. As for experimental spectroscopy methods, the data of a reference run without sample is done in order to normalize the data for each sample with the characteristic features of the source and the box. Loss is experienced only as finite time effect, since only the real part dielectric permittivity is used; due to the finite simulation time, radiation can still be trapped in the structure. Therefore the simulation has to run for a sufficiently long time and 1,000 timesteps have proven to suffice. For low frequencies (long wavelengths), the simulations do not converge and results only above about 0.1c/a are regarded as physically reasonable. At high frequencies (short wavelengths), the noise due to a finite resolution becomes larger and a compromise between finite computational expense and spatial accuracy needs to be found. A resolution in the range of $15\;a^{-1}$ to $20\;a^{-1}$ has proven to be a reasonable value. Note that the reflectance calculated this way includes all intensity that is redirected in general backward direction towards the source, including those scattered beside the Pointing-vector of the original plane wave. Thus, *R* measures the *hemispherical* reflection and *T* measures the *hemispherical* transmission.

## Supplementary Material

pgae383_Supplementary_Data

## Data Availability

The volumetric dataset of the (100) and ($1\bar{1}1$) diamond, the stealthy hyperuniform structure and the amorphous structure is uploaded as *.stl file as supplementary material.

## Preprint

This manuscript was posted on a preprint: [doi.org/10.48550/arXiv.2403.08404].
